# Positive practice environments influence job satisfaction of primary health care clinic nursing managers in two South African provinces

**DOI:** 10.1186/1478-4491-12-27

**Published:** 2014-05-15

**Authors:** Pascalia Ozida Munyewende, Laetitia Charmaine Rispel, Tobias Chirwa

**Affiliations:** 1 Centre for Health Policy & Medical Research Council Health Policy Research Group, School of Public Health, Faculty of Health Sciences, University of Witwatersrand, Private Bag X3, Wits 2050 Braamfontein, Johannesburg, South Africa; 2 Division of Epidemiology and Biostatistics, School of Public Health, Faculty of Health Sciences, University of Witwatersrand, Johannesburg, South Africa

**Keywords:** Job satisfaction, Primary health care, Nursing managers, Health workforce, South Africa

## Abstract

**Background:**

Nurses constitute the majority of the health workforce in South Africa and they play a major role in providing primary health care (PHC) services. Job satisfaction influences nurse retention and successful implementation of health system reforms. This study was conducted in light of renewed government commitment to reforms at the PHC level, and to contribute to the development of solutions to the challenges faced by the South African nursing workforce. The objective of the study was to determine overall job satisfaction of PHC clinic nursing managers and the predictors of their job satisfaction in two South African provinces.

**Methods:**

During 2012, a cross-sectional study was conducted in two South African provinces. Stratified random sampling was used to survey a total of 111 nursing managers working in PHC clinics. These managers completed a pre-tested Measure of Job Satisfaction questionnaire with subscales on personal satisfaction, workload, professional support, training, pay, career prospects and standards of care. Mean scores were used to measure overall job satisfaction and various subscales. Predictors of job satisfaction were determined through multiple logistic regression analysis.

**Results:**

A total of 108 nursing managers completed the survey representing a 97% response rate. The mean age of respondents was 49 years (SD = 7.9) and the majority of them (92%) were female. Seventy-six percent had a PHC clinical training qualification. Overall mean job satisfaction scores were 142.80 (SD = 24.3) and 143.41 (SD = 25.6) for Gauteng and Free State provinces respectively out of a maximum possible score of 215. Predictors of job satisfaction were: working in a clinic of choice (RRR = 3.10 (95% CI: 1.11 to 8.62, *P* = 0.030)), being tired at work (RRR = 0.19 (95% CI: 0.08 to 0.50, *P* = 0.001)) and experience of verbal abuse (RRR = 0.18 (95% CI: 0.06 to 0.55, *P* = 0.001).

**Conclusion:**

Allowing nurses greater choice of clinic to work in, the prevention of violence and addressing workloads could improve the practice environment and job satisfaction of PHC clinic nursing managers.

## Background

There is now global recognition of the importance of the health workforce to health systems development and the achievement of good population health outcomes [1,2]. In many countries there is a health workforce shortage, characterized by inadequate production, suboptimal recruitment, and poor retention and staff management [3-5]. The World Health Organization (WHO) projects that there will be a shortage of 12.9 million healthcare workers by 2035 [6]. More than four million health workers are needed to address the health worker shortages in sub-Saharan Africa, with 11% of the world’s population and only 3% of health workers [7]. Job satisfaction influences health worker motivation, staff retention and performance, which in turn impact on the successful implementation of health system reforms [8]. The global emphasis on universal health coverage has put the spotlight on the revitalization of primary health care (PHC), particularly in resource constrained settings.

In South Africa, key health policies since democracy in 1994 have placed PHC as the cornerstone of the transformation of the national health system [9]. PHC service delivery in South Africa is primarily government-funded, and the more than 1,000 clinics throughout the country are government-owned. The PHC clinics offer a comprehensive range of preventive, promotive and basic curative services that are free at the point of contact [10]. In these clinics, nurses constitute the majority of the health workforce and they are the gatekeepers of the PHC system [11,12]. In 2010, the South African government announced the re-engineering of PHC consisting of: community-based outreach teams and school health services led by nurses; and district clinical specialist teams in which nurses play a key role. A year later in 2011, the government green paper on National Health Insurance [13] was released, laying the foundation for health system reforms towards universal coverage and with PHC as a central tenet of the proposed reforms. PHC is envisaged as nurse-based, supported by doctors and other categories of health professionals. In light of renewed government commitment to PHC reforms in South Africa, this study was conducted to contribute to the development of solutions to the challenges faced by the nursing workforce. Specific challenges at the PHC level include: changing disease burden; mal-distribution of resources between hospitals and clinics; suboptimal supervision and support, rising community expectations and demands [14]. The challenges are exacerbated by insufficient preparation and training of nurses for community-based work [15]. The objective of this study was to determine the job satisfaction of PHC clinic nursing managers in two South African provinces and the predictors of their job satisfaction.

### Job satisfaction and nurses

Job satisfaction is defined as the perceived relationship between what one expects and obtains from one’s job and how much importance or value is attributed to the job [16]. It represents the degree to which employees enjoy their jobs, an important aspect for both the employees and their organizations [17]. In recent years, there has been increased scholarly attention on the job satisfaction of health care professionals because of the need to improve the quality of care, health outcomes and the performance of the health system [18]. A high correlation has been found between employee performance and their job satisfaction [19], while dissatisfaction can be costly and disruptive to organizational effectiveness [20]. Poor levels of job satisfaction are fuelling the health workforce shortages and intensifying nursing imbalances [21].

A large body of literature exists about the job satisfaction of nurses in high-income countries such as Australia [22], Canada [23], China [24], the United States of America and the United Kingdom [25,26]. Increasingly, researchers in low-and middle-income countries (LMICs) have explored job satisfaction and its determinants. These studies have included: job satisfaction and its relationship to intention to leave in Tanzania, Malawi, and South Africa [27], and job satisfaction and turnover in rural South Africa and Papua New Guinea [28,29].

The predictors of nurses’ job satisfaction are complex. Nursing studies show a high correlation between workload, exhaustion, absenteeism, staff conflict and the job satisfaction of health care workers [30] while a strong association also exists between job satisfaction, work commitment and performance [31]. In South Africa, job satisfaction also varied by nursing category, age and gender and is influenced by workplace and the practice environment [18,32-35]. Individual demographic factors, the nature of tasks, pay and relationships with colleagues and the context of work can also influence job satisfaction [29,36,37].

Other research studies amongst nurses have found that the factors that influence job satisfaction are related to the working environment which influences the delivery of quality patient care in PHC clinics [38-40]. Determining job satisfaction among PHC clinic managers and the predictors of such job satisfaction can inform health workforce management strategies and can contribute to the creation of positive practice environments.

### Measure of job satisfaction

Job satisfaction has many explanatory variables and it can be measured through single item questions such as overall satisfaction with one’s job or multiple item measurements relating to co-workers, work demands, job content, workload, skills and pay [41-43]. Although there are a variety of job satisfaction questionnaires in the field of public health and industrial psychology, only a few of these instruments have shown both high reliability and high validity [44]. The Measure of Job Satisfaction (MJS) developed by Traynor and Wade meets both these criteria of high reliability and high validity [45]. The tool consists of seven subscales, listed below.

*Personal satisfaction* (six questions) relates to the extent that nurses feel that they provide good quality patient care, whether their job is personally rewarding, is interesting, challenging and provides opportunities to practise skills while the subscale on *satisfaction with workload* (eight questions) looks at the time available to get through the work, time available for patient care, time spent on administration, staffing levels and overall workload. *Satisfaction with professional support* (eight questions) examines whether managerial and collegial support is available as well as the necessary supervision and guidance required in the role. It also looks at whether one has the opportunity to discuss one's concerns, the presence of teamwork and feeling valued. *Satisfaction with training* (five questions) assesses whether nurses have opportunities for further training, have access to in-service courses, time off or have funding to attend courses and the adequacy of training for the role performed while in the job. S*atisfaction with pay* (four questions) and *prospects* (six questions) are dimensions that deal with pay, grading, career prospects and job security and the last subscale, *satisfaction with standards of care* (six questions) focuses on perceptions of care given to patients. Each of the questions is measured on a Likert scale where 1 = very dissatisfied and 5 = very satisfied [45]. The last question on overall job satisfaction can be added up based on the above subscales to give an overview of overall job satisfaction [45].

## Methods

### Study sites and setting

This was a cross-sectional study conducted among professional nurses working as managers in PHC clinics situated in two South African provinces, namely Gauteng (GP) and Free State (FS), during 2012. GP is an urban province and the economic powerhouse of South Africa with a population of 12.2 million [46], while FS is largely a rural and agricultural province with a population of 2.7 million [45]. Geographical proximity to the researchers, health authority approval and budgetary constraints informed the selection of these provinces.

### Study population and sampling

Professional nurses possessing a four-year qualification and managing eight-hour (day) PHC clinics constituted the population of interest. A PHC clinic nursing manager is a professional nurse in charge of a PHC clinic and is responsible for the overall management and administration of the clinic, including management of staff, health service delivery, patient care, equipment, pharmaceuticals, and supplies [47]. The exclusion criteria were professional nurses managing 24-hour health centres, satellite clinics and those that operate during the weekend or clinics managed by non-nursing managers and clinics that were linked to hospitals; so-called gateway clinics. In order to calculate the sample size, the variable of interest was the proportion of PHC nursing managers that were satisfied with their jobs. This was estimated at 50%, with an assumed 10% precision. Setting the level of significance at 5% and a confidence interval of 95%, a minimum sample size of 96 PHC nursing managers was required (calculated using EpiInfo, Version 6, Centre for Disease Control and Prevention, Atlanta, GA, USA). We included an additional 15 PHC nursing managers to cater for 10% refusals, spoiled or incomplete questionnaires. Following this sample size calculation, stratified random sampling was done by district to select PHC managers from a list of eight-hour (day) clinics in both provinces proportional to each of the health districts (five in GP and five in FS). The final sample consisted of 111 nursing managers, each responsible for a unique clinic.

### Data collection and study variables

The nursing managers were contacted during July 2012 to enlist their participation and to plan the date for fieldwork. At each clinic, an introductory meeting was held with the PHC nursing manager to explain the study components. After obtaining informed consent, a self-administered MJS questionnaire was given to the nurse manager for completion. The research was conducted between July and November 2012. The MJS was used in its original form, but expanded to include demographic factors (age, sex, marital status, number of children, and number of dependants that they were financially responsible for, number of years qualified as a professional nurse, whether the respondent was working in a clinic of their choice, PHC clinical training and any current studies). The instrument also collected information on the practice environment, particularly concern about violence, experience of any form of violence, perpetration of violence by self, colleagues, patients or relatives, feelings of exhaustion while at work, taking sick leave when actually not sick, intention/desire to leave their employer in the next year and awareness of the PHC re-engineering reforms. These variables were added following key informants’ recommendations obtained during formative, qualitative research conducted prior to the survey. We also included an open-ended question to allow clinic managers to comment on any aspect related to their job satisfaction. The questionnaire took ten minutes to complete. We used the MJS subscales and thematic content analysis [48] to analyze the open-ended responses. The themes helped in organizing, managing, interpreting and retrieving meaningful segments of quotes and linking segments of text to a particular subscale of job satisfaction [49]. Two other researchers read the responses independently to ensure reliability and to establish inter-coder agreement [49,50].

We pre-tested the tool with three PHC nursing managers prior to implementation and no adjustments were necessary. These three respondents were excluded from the main study.

### Ethical considerations

We obtained ethics approval from the University’s Human Research Ethics Committee (M 12 02 48). All relevant health care authorities granted permission for the study. In the field, PHC clinic nursing managers were given a detailed information sheet, as well as verbal explanation of the study, the voluntary, confidential and anonymous nature of participation and the sampling methodology of the research. We ensured anonymity of responses and maintained confidentiality of the data by ensuring that survey results would not be tied back to an individual person or individual PHC facility.

### Data management and analysis

The data were analyzed using Stata version 12 (StataCorp, College Station, TX, USA). A descriptive analysis of socio-demographic characteristics and work (practice) environment factors was conducted, using frequency tabulations and summary measures.

Two approaches were used to analyze the data so as to understand job satisfaction levels and its predictors. The first one involved the use of mean scores for each of seven subscales of job satisfaction as described above [45]. The mean scores were calculated by dividing the sum of subscale score by the number of questions in that subscale. The seven subscale scores were then aggregated to provide an overall job satisfaction mean score, which is the overall mean score was calculated from all the 43 questions that form the seven subscales of job satisfaction in the MJS tool. The possible scores for overall job satisfaction range from 43 to 215.

The second approach involved determining job satisfaction predictors through regression modelling. For this approach, the five point Likert scale of overall job satisfaction was categorized into three categories (satisfied = scores 4 and 5, indifferent = score 3 and dissatisfied = scores 1 and 2). Multinomial logistic regression was then used to determine predictors of job satisfaction in the two provinces. The reference group for job satisfaction and dissatisfaction was the ‘indifferent’ category. The regression models were fitted separately for GP and FS. It was decided in advance that a combined model would be fitted only if no significant differences existed in job satisfaction between the two provinces. All variables found significant (with *P* < 0.25) at the univariate analysis were included in the multinomial logistic regression. All tests were conducted at 5% significance level.

### Validity and reliability

After piloting, to determine validity and reliability of the MJS instrument, Cronbach’s alpha (CA) coefficients were used to assess the reliability of the scale and 0.94 or (94%) was obtained, signifying that it had high internal consistency [44]. The lowest CA was 0.75.

## Results

### Demographic characteristics of primary health care nursing managers

Table 1 indicates the socio-demographic and work environment characteristics of study participants. A total of 111 questionnaires were distributed to clinic nursing managers in GP and FS provinces. A total of 108 questionnaires were returned (GP n = 62 and FS n = 46) representing a survey response rate of 97%. The mean age of participants was 49 years (SD: 7.8). The majority in both provinces were female (92%); married (63%); employed in a permanent position (89%); and working in a clinic of choice (59%). Slightly over one third of the respondents (36%) had a professional nursing experience of between 21 and 30 years. The majority (76%) had formal qualifications in PHC clinical management and almost one third (29%) indicated that they were not aware of the PHC re-engineering reforms in South Africa.

**Table 1:** Socio-demographic and practice environment characteristics of study participants (n = 108)

**Characteristics**	**GP n (%)**	**FS n (%)**	**Total n (%)**
Total	62 (57.4)	46 (42.6)	108 (100.0)
Age (years)			
21-30	3 (4.84)	0	3 (2.78)
31-40	10 (16.1)	4 (8.70)	14 (12.9)
41-50	17 (27.4)	24 (52.1)	41 (37.9)
51+	32 (51.6)	18 (39.1)	50 (46.3)
Sex			
Male	7 (11.3)	2 (4.35)	9 (8.33)
Female	55 (88.7)	44 (95.6)	99 (91.7)
Marital status			
Married	42 (67.7)	26 (56.5)	68 (62.9)
Living together	1 (1.61)	1 (2.17)	2 (1.85)
Single	8 (12.9)	8 (17.4)	16 (14.8)
Divorced	4 (6.45)	7 (15.22)	11 (10.2)
Widowed	7 (11.3)	4 (8.70)	11 (10.2)
PHC qualification			
No	14 (22.9)	12 (26.1)	26 (24.1)
Yes	48 (77.4)	34 (73.9)	82 (75.9)
Years qualified as a professional nurse			
0-10	11 (17.7)	4 (8.70)	15 (13.9)
11-20	15 (24.2)	21 (45.6)	26 (33.3)
21-30	22 (35.5)	17 (36.9)	39 (36.1)
31-40	14 (22.3)	4 (8.70)	18 (16.7)
Clinic choice			
No	27 (43.5)	17 (36.9)	44 (40.7)
Yes	35 (56.5)	29 (63.0)	64 (59.3)
Permanent position			
No	8 (13.8)	3 (6.52)	11 (10.6)
Yes	50 (86.2)	43 (93.5)	93 (89.4)
Number of dependents			
0	3 (4.84)	1 (2.17)	4 (3.70)
1-4	54 (87.1)	42 (91.3)	96 (88.9)
5-8	5 (8.06)	3 (6.52)	8 (7.41)
Currently studying			
No	47 (75.8)	40 (86.9)	87 (80.6)
Yes	15 (24.1)	6 (13.0)	21 (19.4)
Sector			
Provincial	16 (25.8)	46 (100.0)	62 (57.4)
Local authority	46 (74.2)	0	46 (42.6)
Concerned about violence in the workplace			
No	36 (58.1)	25 (54.4)	61 (56.5)
Yes	26 (41.9)	21 (45.6)	47 (43.5)
Ever experienced violence in the workplace in the last year (either from colleague or patient)			
No	40 (65.6)	33 (73.3)	73 (68.8)
Yes	21 (34.4)	12 (26.7)	33 (31.1)
Suffered verbal abuse in the last year			
No	38 (61.3)	28 (60.8)	66 (61.1)
Yes	24 (38.7)	18 (39.1)	42 (38.9)
Verbally abused or shouted at someone in the workplace in the last year			
No	56 (91.8)	43 (93.5)	99 (92.5)
Yes	5 (8.20)	3 (6.52)	8 (7.5)
Felt humiliated or shamed by another in the workplace in the last year			
No	47 (75.8)	37 (80.4)	84 (77.8)
Yes	15 (24.2)	9 (19.6)	24 (22.2)
Felt too tired to work while on duty			
No	28 (45.2)	24 (52.2)	52 (48.2)
Yes	34 (54.8)	22 (47.8)	56 (51.8)
Taken sick leave when actually not sick			
No	59 (95.2)	46 (100.0)	105 (97.2)
Yes	3 (4.84)	0	3 (2.78)
Intention to leave employer			
No	54 (87.1)	34 (73.9)	88 (81.5)
Yes	8 (12.9)	12 (26.1)	20 (18.5)
Awareness of PHC revitalization currently underway			
No	22 (36.1)	9 (19.6)	31 (28.9)
Yes	39 (63.9)	37 (80.4)	76 (71.0)

### Practice environment factors

The majority of participants (97%) did not take sick leave while not sick; 98% had not been absent from work without authorization and 81% indicated that they did not intend to leave their workplace in the year following the survey. However, 43% of PHC clinic nursing managers indicated that they were concerned about violence in the workplace, 31% had experienced violence; and 39% had experienced verbal abuse from other colleagues, patients or relatives in the workplace. More than half of the study participants (52%) reported that they felt ‘too tired to work’ while on duty.

### Job satisfaction scores based on the subscales

Table 2 lists the seven subscales of job satisfaction for PHC nurse managers in GP and FS. The overall mean score of job satisfaction for nurse managers in GP (142.8; SD **±** 24.3) and FS (143.4; SD **±** 25.6) were similar, although these differences were not statistically significant (*P* = 0.90).

**Table 2:** Mean scores of job satisfaction subscale individual questions by province

**Assessment subscales of job satisfaction**	**GP (n = 62)**	**FS (n = 46)**	**Total possible score**	***P*-value**
	$\overset{¯}{x}$ **± SD**	$\overset{¯}{x}$ **± SD**		
**Personal satisfaction**				
The feeling of worthwhile accomplishment I get from my work	3.29 ± 1.08	3.26 ± 0.97		
The amount of personal growth and development I get from my work	3.65 ± 0.85	3.46 ± 1.09		
The extent to which my job is varied and interesting	3.69 ± 0.94	3.76 ± 0.10		
The amount of independent thought and action I can exercise in my work	3.61 ± 0.96	3.72 ± 0.96		
The extent to which I can use my skills	4.05 ± 0.69	3.65 ± 0.10		
The amount of challenge in my job	3.38 ± 1.07	3.28 ± 0.98		
**Total score**	21.59 ± 3.91	21.13 ± 3.77	30	0.535
**Satisfaction with workload**				
The time available to get through my work	2.45 ± 1.05	2.26 ± 1.06		
The amount of time spent on administration	2.61 ± 0.99	2.21 ± 0.89		
My workload	2.38 ± 1.10	2.19 ± 1.24		
Overall staffing levels	2.35 ± 1.17	2.37 ± 1.12		
The amount of time available to finish everything that I have to do	2.45 ± 1.00	2.15 ± 0.97		
What I have accomplished when I go home at the end of the day	3.26 ± 1.05	3.11 ± 1.08		
The hours I work	3.71 ± 0.95	3.76 ± 0.92		
The time available for patient/client care	3.10 ± 1.09	2.87 ± 1.15		
**Total score**	22.16 ± 5.77	20.83 ± 6.37	40	0.258
**Satisfaction with professional support**				
The degree to which I feel part of a team	3.94 ± 0.67	0.87 ± 0.96		
The opportunities I have to discuss my concerns	3.26 ± 0 .93	3.59 ± 1.12		
The amount of support and guidance I receive	3.35 ± 0.99	3.56 ± 1.00		
The people I talk to and work with	3.92 ± 0.68	4.24 ± 0.64		
The degree of respect and fair treatment I receive from my boss	3.61 ± 0.96	3.50 ± 1.09		
The support available to me in my job	3.32 ± 1.04	3.37 ± 0.10		
The overall quality of the supervisions I receive in my work	3.61 ± 0.84	3.56 ± 0.86		
The contact I have with colleagues	3.97 ± 0.77	4.09 ± 0.72		
**Total score**	28.92 ± 4.77	29.78 ± 5.38	40	0.380
**Satisfaction with training**				
Being funded for courses	3.45 ± 1.20	2.98 ± 1.20		
The opportunities I have to advance my career	3.63 ± 0.85	3.28 ± 1.00		
The extent to which I have adequate training for what I do	3.71 ± 0.80	3.76 ± 0.77		
Time off for in-service training	3.53 ± 1.05	3.13 ± 1.17		
The opportunity to attend courses	3.60 ± 1.05	3.43 ± 1.19		
**Total score**	17.85 ± 3.40	16.50 ± 3.06	25	0.035
**Satisfaction with pay**				
Payment for the hours I work	2.79 ± 1.33	3.76 ± 0.97		
My salary/pay scale	2.69 ± 1.36	3.80 ± 0.96		
The degree to which I am fairly paid for what I contribute to this organization	2.77 ± 1.26	3.43 ± 1.11		
The amount of pay I receive	2.63 ± 1.33	3.52 ± 1.09		
**Total score**	10.73 ± 4.73	14.43 ± 3.72	20	0.000
**Satisfaction with prospects**				
My prospects for promotion	3.18 ± 0.95	3.15 ± 0.99		
My prospects for continued employment	3.50 ± 0 .90	3.59 ± 1.05		
The amount of job security I have	3.65 ± 1.01	3.00 ± 1.21		
The possibilities for a career in my field	3.66 ± 0.83	3.59 ± 1.02		
The outlook for my professional group/branch of nursing	3.53 ± 0.90	3.39 ± 0.80		
How secure things look for me in the future of this organization	3.42 ± 1.06	3.30 ± 1.09		
**Total score**	20.87 ± 3.52	19.89 ± 4.28	30	0.195
**Satisfaction with standards of care**				
The quality of work with patients/clients	3.18 ± 0.98	3.15 ± 1.11		
The standard of care given to patients/clients	3.42 ± 1.02	3.41 ± 1.17		
The way that patients/clients are cared for	3.58 ± 0.84	3.63 ± 1.04		
The standard of care that I am currently able to give	3.60 ± 0.93	3.63 ± 0.93		
The general standard of care given in this unit	3.44 ± 0.97	3.54 ± 0.96		
Patients are receiving the care that they need	3.47 ± 0.97	3.48 ± 1.11		
**Total score**	20.68 ± 4.52	20.85 ± 5.00	30	0.85
**Overall satisfaction**	**142.80 ± 24.3**	**143.41 ± 25.6**	215	0.90

There were statistically significant differences between the mean scores for satisfaction with training in GP (17.85; SD **±** 3.40) where scores were higher than in the FS (16.50; SD **±** 3.06) (*P* = 0.03). Within this subscale, time available to attend courses, opportunities to advance their careers and the extent to which they had adequate training for their job was higher in GP than in FS. PHC nurse managers in FS (14.43; SD **±** 3.72) had a significantly higher mean score for satisfaction with pay than those in GP (10.73; SD **±** 4.73 (<0.001), who were mostly satisfied with the pay and payment for the hours worked. The mean scores for the subscales on personal satisfaction, professional support, prospects and standards of care were relatively high in both provinces, and the differences were not statistically significant.

### Nurses’ comments on job satisfaction

All nursing managers responded to the open-ended question; ‘*what do you think can be done to improve your job satisfaction in this clinic?*’

### Personal satisfaction and prospects

Nursing managers had relatively high mean scores on personal satisfaction in GP (21.59; SD ± 3.91) and in FS (21.13; SD ± 3.77) compared to satisfaction with career prospects (GP 20.87; SD ± 3.52 and (FS 19.89; SD ± 4.28). However, the qualitative comments made by nursing managers contradicted these high scores on personal satisfaction and career prospects. Their comments show that nursing managers were affected by their working conditions, which limited their ability to practise their skills. These challenging working conditions included: the lack of clinic maintenance (broken windows, leaking ceilings, dysfunctional sewage systems); the poor state of clinic infrastructure (insufficient consultation rooms and lack of patient waiting areas); and the unavailability of basic or clinical equipment (working refrigerators, blood pressure machines and telephones). The comments below are illustrative of this disjuncture between quantitative scores and qualitative perceptions.

‘*We want to have working equipment that is sustainable for a long period. An improved communication system that is not dependent on manual writing and messengers to deliver information is needed. I need a working environment and structure that is conducive and welcoming to both staff and patients.*’ (Respondent # 93, GP)

Another nursing manager said:

‘*I feel so discouraged…In fact I feel I should leave nursing and pursue other new ventures…*’ (Respondent # 42, FS)

### Workload

Nursing managers from both provinces had low scores on workload (GP 22.16; SD ± 5.77 and FS 20.83; SD ± 6.37). These low scores were borne out by the qualitative responses, which highlighted work pressure, exacerbated by shortages of health professionals (nurses, doctors, social workers, pharmacists) and support staff (cleaners, gardeners, security guards). The following excerpts reflect how these issues affected patient care and their ability to manage the clinics.

‘*At the moment, the clinic manager must wear all the caps, for example when the cleaner is not available she must clean the clinic, when the pharmacy assistant is not available she must dispense medication. If we can have relief staff in all categories to close the gap when there is shortage that would be nice because in PHC, staffing is important for quality service.*’ (Respondent # 9*,* FS)

‘*We need to have more staff, especially nurses as we render a lot of services to our patients*, *the workload becomes unbearable*.’ (Respondent *#* 82*,* GP)

### Professional support

Nursing managers had high mean scores for professional support in GP (28.92; SD ± 4.77) and in FS (29.78; SD ± 5.38). However, the qualitative responses revealed that nursing managers experienced a general lack of support from their supervisors who either interfered or blamed them for wrong doing. Clinic managers reported lack of consultation on decision-making, especially those related to financial management. In addition, nursing managers did not feel that they had the authority to manage their clinics. One said:

‘*Recognition and degree of support from my senior manager is non*-*existent*…*the big boss does not recognize my contribution in the health system* - *he is a fault finder and very quick to blame and judge when things are not done. The management needs to consider my personal point of view*…’ (Respondent *#* 82*,* GP)

‘*We need to get support from our supervisors because patients are disrespecting us*…*our communities have to know that rights go hand in hand with responsibility. Our supervisors must learn to respect us people on the ground because we are the ones doing the spade work. When there is a problem they must ask for both sides of the story*, *not only to get the patient*’*s side and always thinking the staff is always wrong.* (Respondent *#* 49*,* FS).

### Training

Although nursing managers in both provinces had low scores on satisfaction with training, the scores for FS was lower (16.50; SD ± 3.06) compared to GP (17.85; SD ± 3.40), and this difference was statistically significant (*P* = 0.035). They expressed a desire for continuing professional development and keeping abreast of new developments in nursing. They also indicated a need for training in information technology, including the use of computers. They were of the opinion that computers would cut down on the paper work and assist with the completion of different patient registers.

‘*The clinic manager should also be sent to school to upgrade themselves about new developments in nursing and management but in our region only nurses* (not managers) *are capacitated. If you apply for study leave as a manager our seniors will ask you who is going to manage the clinic*…’ (Respondent *#* 54*,* GP*)*

‘*Take me to training courses*, *for example computer courses and managerial skills*.’ (Respondent *#* 1*,* FS)

### Pay

PHC nursing managers in GP had low scores on the satisfaction with pay (10.73; SD ± 4.73) compared to those in FS (14.43; SD ± 3.72). This was supported by the qualitative comments, which illustrated that nursing managers were of the opinion that their salaries were not commensurate with the increasingly complex disease burden, growing number of patients, and various health system reforms. All these issues resulted in increased management responsibilities at the PHC level.

‘*The clinic manager has lots of responsibilities but they are underpaid*, *we get the same salaries as professional nurses and nursing assistants*…’ (Respondent *#* 54*,* GP)

Nursing managers also complained that poor salaries for increased responsibilities were made worse by an unfair performance management and reward system:

‘*The performance management development system must be reviewed. Staff members who qualify for a bonus are not getting it. The system of rewarding a certain percentage of staff is not working*; *instead*, *it is creating bad relations between the manager and the staff*…’ (Respondent *#* 36*,* FS)

### Standards of care

Although nursing managers had high scores on satisfaction with standards of care (GP 20.68; SD ± 4.52; FS 20.85; SD ± 5.00), some complained about the large numbers of patients at PHC facilities. They were of the opinion that services had expanded, exacerbated by increasing numbers of patients from different catchment areas and neighbouring countries without the additional staff. Managers indicated that quality of care could be improved with adequate staff to cover disease programmes:

‘*Levels of staffing should be increased to improve the standard of care to clients. Currently*, *the demands of the work and the number of clients seen by each nurse compromises quality of care in terms of support services. Support services for HIV Counselling and Testing were provided when the ARV programme started. But due to non*-*payment of these counsellors* (lay health workers), *support services were removed and this exacerbates the work load and demands placed on the nursing staff. It is not possible to have job satisfaction when you are constantly under pressure to do more with inadequate resources*…’ (Respondent *#* 79*,* GP)

Filling in too many disease programme registers was a source of tension as it diverted time needed for patient care:

‘…*we have just started the HIV*/*AIDS antiretroviral* (*ARV*) *programme. There is too much paper work that prohibits us to from doing proper nursing care*…’ (Respondent *#* 36*,* FS)

### Security

Security in the clinics was a big concern for nursing managers who felt that their workplaces were unsafe. They mentioned that most of their clinics do not have alarm systems or security personnel to guard the facilities. Examples were given of burglaries where equipment, drugs and personal belongings were stolen or damaged. Nursing managers also cited threats of violence from patients and from the community. Basic security infrastructure such as a security fence or availability of alarms, having phones that work in case of an emergency were requested and thought to be essential to improve job satisfaction:

‘*A security service for clinic staff is needed. We had a break in from the community and they actually wanted to force entry and we have no security available around the premises. They were having protest marches against the municipality but they also threatened us with war*…’ (Respondent # 42, FS)

‘*Clients are demanding services which are promised to them by politicians and it is not always possible to live up to these expectations. This leads to constant abuse from some clients and at times being threatened with physical violence as a result of this.*’ (Respondent *#* 79*,* GP)

### Predictors of job satisfaction

Tables 3 and 4 show the results of the univariate and multiple logistic regressions respectively. The predictors of job satisfaction based on the multiple logistic regression analysis were: working in a clinic of choice, feeling too tired and being verbally abused at work by patients, colleagues or someone close to the manager.

**Table 3:** Multinomial univariate logistic analysis outcome = job satisfaction

**Predictor**	**Dissatisfied compared to indifferent group**			**Satisfied compared to indifferent group**		
	*RRR	**95% CI**	***P*-value**	*RRR	**95% CI**	***P*-value**
Tired at work						
No						
Yes	2.57	0.64-10.3	0.18	0.34	0.13-0.93	0.037
Verbal abuse						
No						
Yes	0.56	0.16-1.91	0.35	0.14	0.05-0.42	0.001
Clinic of choice						
No						
Yes	0.83	0.25-2.74	0.761	1.08	0.40-2.90	0.087
Years qualified as PN						
0-10						
11-20	2	0.14-28.41	0.609	0.66	0.11-3.81	0.649
21-30	2.75	0.21-35.83	0.440	0.41	0.07-2.29	0.315
31-40	1.33	0.08-20.10	0.835	0.22	0.03-1.38	0.108
Province						
GP	…					
FS		0.75-8.78	0.132	1.56	0.56-4.35	0.393
Sex						
Male						
Female	2.09	0.17-25.00	0.557	0.96	0.18-5.18	0.968
Marital status						
Married	…	…	…	…	…	…
Living together	0.93	0.61-2.26	1.00	0.27	…	0.99
Single	0.37	0.08-4.31	0.282	0.57	0.16-2.00	0.38
Divorced	0.62	0.36-37.70	0.628	0.63	0.13-2.89	0.56
Widowed	3.71	0.50-2.29	0.267	1.90	0.20-17.28	0.57
Age						
21-30						
31-40	..	…	0.988	0.50	0.07-16.30	0.931
41-50	..	…	0.987	0.61	0.14-22.68	0.655
> 50	..	…	0.987	0.37	0.11-17.16	0.793
Permanent position						
No						
yes	1.42	0.21-9.51	0.717	1.47	0.33-6.47	0.608
PHC qualification						
No						
Yes	0.80	0.22-2.94	0.741	1.47	0.48-4.50	0.499
Currently studying						
No						
Yes	1.57	0.24-10.51	0.637	3.33	0.70-5.85	0.130

Legend: *RRR, relative risk ratio.

**Table 4:** Final multivariate multiple logistic regression model

**Predictor**	**Dissatisfied**			**Satisfied**		
	*RRR	**95% CI**	***P*-value**	*RRR	**95% CI**	***P*-value**
Tired at work						
No	1.00	Reference		1.00	Reference	
Yes	4.89	3.56-35.89	0.151	0.19	0.08-0.50	0.001
Verbal abuse						
No	1.00	Reference		1.00	Reference	
Yes	2.22	0.50-9.82	0.291	0.18	0.06-0.545	0.002
Clinic of choice						
No	1.00	Reference		1.00	Reference	
Yes	0.41	0.94-1.83	0.244	3.10	1.11-8.62	0.030

Legend: *RRR, relative risk ratio.

Outcome = job satisfaction (relative to reference category).

Among study participants who worked in a clinic of choice, the relative risk ratio (RRR) of them being satisfied with their job increases by 3.10 times compared to those in the indifferent group (95% CI 1.11 to 8.62, *P* = 0.030). For participants who were too tired at work, the RRR of them being dissatisfied with their jobs increases by 4.9 times compared to the indifferent group (95% CI 3.56 to 35.89, *P* = 0.151). Among study participants who experienced verbal abuse while at work, the RRR of them being dissatisfied with their job is 2.22 times more likely compared to those in the indifferent group (95% CI: 0.50 to 9.82, *P* = 0.291).

## Discussion

We conducted a cross-sectional survey to determine the job satisfaction of PHC clinic nursing managers in two South African provinces. Our study is one of the first African studies that uses the MJS tool to examine the job satisfaction of nursing managers at the PHC clinic level, but that combined it with socio-demographic variables and that explored the role of workplace violence in job satisfaction. The tool was designed specifically for nurses working in community settings, it has high validity and reliability and it focuses on all aspects of job satisfaction. The tool is useful and relevant for application at PHC (or community) settings in other parts of South Africa and in other low- and middle-income countries (LMICs). As the MJS tool was originally designed and used in the UK, it has applicability to community-based settings in general.

We have found that these managers had moderate levels of job satisfaction. The findings are similar to those of two other studies done in rural KwaZulu-Natal, South Africa and in the United Kingdom [45,51] that used the same MJS tool with non-nursing managers. A study in Egypt in 2006 found moderate job satisfaction levels amongst PHC nurses [52]. Although our study population consisted of nurses in charge of PHC clinics, the findings are also similar to two other studies in Palestine and South Africa that focused on nurses working in PHC settings [53,54]. A multi-country study on job satisfaction among different categories of health workers (including nurses) found low job satisfaction and high intention to leave the workplace among South African nurses, with 41% actively seeking other jobs [27]. However, a different instrument was used in this study, so the results may not be comparable.

### Age, tenure and job satisfaction

The mean age of nurses in our study was 49 years. A similar study in South Africa using the same MJS tool also found that a large number of respondents were between the ages of 41 and 50 years, highlighting an ageing nursing workforce that has been pointed out by others [51,55,56]. Our study found that the job satisfaction of clinic nursing managers decreased for every unit increase in years qualified as a nurse and as they got older. This is similar to the finding of another South African study that found an association between years of nursing experience and job satisfaction [51]. The findings of our study are also similar to those of a Jordan hospital study, which found that older nurses were leaving the profession before retirement due to low levels of job satisfaction associated with poor working conditions [57]. Similarly, an international study on hospital nursing unit managers found that tenure (or years in nursing) had a negative relationship with job satisfaction [58]. The negative relationship between tenure and job satisfaction for our study respondents could be related to the comments they made about increasing workloads, roles and responsibilities of clinic managers due to ongoing health system reforms.

### Job satisfaction subscales

The subscales with the highest scores were personal satisfaction, professional support, training, prospects and standards of care, while the lowest scores were obtained for workload and pay (Table 2). Although there were differences in the mean scores, the variation could be due to the small sample size. Another South African study also found that nurses were satisfied about their personal contribution to work and least satisfied with pay and prospects [51]. In contrast, a study among district nurses in the UK using the same instrument found high scores on personal satisfaction, work load and satisfaction with pay and prospects [59].

PHC nursing managers in GP had low mean scores on payment for the hours worked and the degree to which they felt they were fairly rewarded for their contributions in the clinics where they work. PHC nurse managers in FS had a significantly higher mean score for satisfaction with pay than those in GP (*P* = 0.001). This could be related to the higher cost of living in the urban GP, compared to FS and because nurse managers in GP had more dependants than those in FS (*P* = 0.042). Dissatisfaction with pay has also been found in other studies on job satisfaction in the United Kingdom [59] and in South Africa [60].

Our study found that PHC clinic nursing managers, especially those in the FS, scored high on satisfaction with professional support. However, there is a disjuncture between the scores and the comments made by these nurses. This is also in contrast to the findings of other studies that have found weak supervision at PHC level [51,61], and that supervisors in LMICs lack leadership and management skills [62,63]. As shown in Table 2, some clinic managers in the FS were concerned about the degree to which they felt to be part of a team which suggests that there could be suboptimal teamwork in clinics in the province. Where similar studies have been conducted in hospital settings, the absence of teamwork influenced workloads leading to absenteeism, burnout and low levels of job satisfaction [64,65].

PHC clinic nursing managers in our study had low levels of satisfaction with workload, especially on time available to get through work, on administration and overall staffing levels. Responding to the open ended question about issues that affected their job satisfaction, many clinic managers mentioned that they often work with limited human resources and have extended responsibilities beyond their job descriptions. Another South African study found that appropriate nurse to patient ratios would contribute a great deal to nurse retention, commitment and job satisfaction, which in turn would improve quality of patient care [66]. Concerns about workload are further exacerbated by staff shortages in South Africa and the complex burden of disease (communicable, noncommunicable, perinatal and maternal conditions, and injury-related complications) which have a negative influence on nurses’ job satisfaction [67,68].

### Predictors of job satisfaction

In our study, working in a clinic of choice, being too tired to work while on duty, and concern about violence were predictors of job satisfaction (Table 4). This is similar to the findings of other studies that found that work environment, workload, insufficient time for training and suboptimal management were negatively correlated with job satisfaction [26,53,69,70]. However, the findings are not directly comparable because of different contextual situations and different methodological approaches. Feeling too tired at work [71,72] and suffering verbal abuse both have the potential to reduce the quality of care for patients [73]. We could not find other studies that examined the association between verbal abuse and job satisfaction amongst nurses. The comments made by nurses also pointed to other aspects of the practice environment such as poor clinic infrastructure, lack of functioning equipment, safety concerns, lack of recognition, and suboptimal supervision and management. Nursing managers commented on the lack of security in PHC facilities which compromised their personal safety as well as that of their patients. The International Council of Nursing (ICN) has noted that practice environments that address the challenges of physical and psychological violence and workloads, and that offer professional support and prospects for professional development can influence job satisfaction and individual performance [74]. The ICN has recommended safe and enabling practice environments for nurses at all types of health care facilities [74]. Hence, our study findings add weight to the ICN recommendations on positive practice environments for nurses.

In light of the study finding that highlights the importance of choice of clinic to nurses’ job satisfaction, we recommend that district and executive health managers discuss and consult nurses about their placement. This is relatively easy to address through good management practices and the establishment of staff forums at the PHC level. It is also in line with the recent recommendation at the 2013 Third Global Forum on Human Resources for Health that proposed mechanisms to enhance retention of health workers and to give health workers a voice to make inputs [6]. The findings also point to the need for an enabling practice environment that includes supportive supervision and management, and addressing other concerns such as staff shortages, safety, infrastructure and availability of functioning equipment. Policy-makers are aware of these issues that the study provides with evidence of challenges at the PHC clinic level. Addressing these issues requires additional financial resources and the current health system reforms provide a window of opportunity for concrete action.

Social marketing campaigns that raise awareness about the rights and responsibilities of users and providers will go some way towards addressing the concerns expressed regarding verbal abuse. Importantly, the study findings require dedicated effort to implement the recommendations of the 2013 strategic plan on nurse education, training and practice [55]. This plan contains recommendations on nursing education and training; resources in nursing; governance, leadership, legislation and policy; positive practice environments; and compensation, benefits and conditions of employment [55].

### Health system reforms

Almost one third of our study respondents (29%) indicated that they were not aware of the current PHC re-engineering reforms in South Africa. The open-ended comments suggest that PHC nursing managers feel left out of current health system reforms and feel that information is passed down to them without consultation. Although the notion of job satisfaction is subject to individual interpretations [75] or other cultural biases, the moderate levels of job satisfaction could be linked to a series of reforms in the health system, notably at PHC level. This result is not unique to South Africa as evidence shows that health system reforms in the Africa region [76] or elsewhere [77] tend to expand or extend the roles of health workers, but are often imposed on health workers without their inputs or with insufficient discussion, thereby affecting job satisfaction and/or health service delivery. Existing evidence suggests that shared governance and leadership and health worker inclusion in the development of policies and strategies is an important aspect that positively influences job satisfaction and retention of nursing managers [78].

Policy-makers in all the countries, but particularly those with health workforce shortages in LMICs, should acknowledge that job satisfaction of nursing managers is an important aspect of staff retention. A previous Canadian study among hospital employees that focused on restructuring of hospitals and its effect on job satisfaction reported poor levels of job satisfaction [79]. Other studies have also noted that high levels of job satisfaction are likely to enhance retention, influence the quality of care and contribute to the success of health system reforms towards universal coverage [26,66].

The study yields important information, but the findings are not generalizable to all PHC nursing managers in South Africa. Gauteng is a well-resourced urban province, while Free State has both urban and rural characteristics. Both provinces have relatively good PHC facility infrastructure and staffing levels. This is in contrast to rural provinces of South Africa that suffer from major financial and human resource constraints. Hence, PHC nursing managers in these rural provinces may experience lower levels of overall job satisfaction, and have lower scores on the seven subscales. We were also unable to show statistically significant differences between rural and urban clinic nurse managers because of the relatively small sample size. These limitations are not specific to our study but are a general indication of human resource studies in Africa that are often based on small numbers of health care professionals. Substantial funding is required to obtain bigger and more representative samples to carry out longitudinal studies using the MJS tool that will inform additional and effective health workforce strategies in LMICs.

Nonetheless, there are many study strengths. The use of the MJS tool with high reliability and validity in an African setting and exploring the association between positive practice environments and job satisfaction is novel. The overall job satisfaction scores and for each of the subscales provide a basis for future comparisons with a national, more representative sample of PHC nursing managers, as well as with other LMICs. Combining a pre-tested instrument with the opportunity for *verbatim* comments from participants is a strength and provides insights into the issues that influence the job satisfaction scores in the two provinces. The high response rate in the survey is also a strength and allows for the study findings to be generalized to other nursing managers in the two provinces. The option of a self-administered questionnaire provided participants with greater privacy and is likely to have limited social desirability bias. The concurrent measurement of job satisfaction and the predictors of job satisfaction provide useful information to plan nurse retention strategies in PHC clinics. The research identifies key issues that influence the job satisfaction of PHC nursing managers’, notably working in a clinic of choice; exhaustion (a proxy for workload) and verbal abuse. These issues have not featured high on health workforce policies or strategies, and could be used to inform these, both in South Africa and in other LMICs. At the same time, larger, national representative studies are needed to confirm these predictors of job satisfaction. In light of the envisaged seminal role of nurses in reforms towards universal health coverage, the complex and multiple issues raised by nursing managers would need to be addressed to ensure the successful implementation of these reforms. The study findings will be used for a policy brief requested by an advisor in the South African Ministry of Health.

## Conclusion

There is renewed attention globally on universal health coverage and the importance of the health workforce in achieving this goal. This study highlights the job satisfaction challenges faced by nursing managers at the PHC level. The predictors of nursing managers’ job satisfaction were the ability to choose the clinic to work in, feeling too tired to work whilst at work and experiencing verbal abuse. Our findings highlight the need for policy-makers to create positive practice environments that encourage flexibility and that aim to prevent workplace violence. Staff retention is important to break the cycle of turnover, while additional staffing will deal with complaints about workloads that contribute to feelings of exhaustion among managers. The success of health system reforms, both locally and internationally, are dependent on addressing the challenges faced by the nursing workforce. Our study provides useful information and recommendations in order to make a difference.

## Abbreviations

ARV: antiretroviral; FS: Free State; GP: Gauteng; HIV: human immunodeficiency virus; ICN: International Council of Nurses; LMICs: low- and middle-income countries; MJS: Measure of Job Satisfaction; NHI: National Health Insurance; PHC: primary health care; RESON: Research on the State of Nursing; RRR: relative risk ratio; SD: standard deviation; UK: United Kingdom; USA: United States of America; WHO: World Health Organization.

## Competing interest

The authors declare no conflict of interest, financial or otherwise.

## Authors’ contributions

PM participated in the conceptualisation of the study, led the field work, conducted the literature review, performed the initial analysis, interpreted the results and wrote initial drafts of the manuscript. LCR is Principal Investigator who conceptualised and designed the multi-year research project, and she is PM’s PhD supervisor. LCR contributed to the writing, interpretation of study findings and revision of the manuscript. TC assisted with the analysis of study findings and contributed to the writing and revision of the manuscript. All authors revised, read and approved the final manuscript.
